# Artificial intelligence in chronic kidney diseases: methodology and potential applications

**DOI:** 10.1007/s11255-024-04165-8

**Published:** 2024-07-25

**Authors:** Andrea Simeri, Giuseppe Pezzi, Roberta Arena, Giuliana Papalia, Tamas Szili-Torok, Rosita Greco, Pierangelo Veltri, Gianluigi Greco, Vincenzo Pezzi, Michele Provenzano, Gianluigi Zaza

**Affiliations:** 1 Department of Mathematics and Computer Science, University of Calabria, 87036 Rende, CS Italy; 2 Department of Medical and Surgical Sciences, University of Catanzaro, 88100 Catanzaro, Italy; 3 Nephrology, Dialysis and Renal Transplant Unit, Department of Pharmacy, Health and Nutritional Sciences, University of Calabria, Rende - Hospital ‘SS. Annunziata’, Cosenza, Italy; 4 Department of Computer Science, Modeling, Electronics and Systems Engineering, University of Calabria, 87036 Rende, CS Italy; 5 Division of Nephrology, Department of Internal Medicine, University Medical Center Groningen, Groningen, the Netherlands

**Keywords:** Chronic kidney disease, Artificial intelligence, Machine learning, Deep learning, Explainable artificial intelligence

## Abstract

Chronic kidney disease (CKD) represents a significant global health challenge, characterized by kidney damage and decreased function. Its prevalence has steadily increased, necessitating a comprehensive understanding of its epidemiology, risk factors, and management strategies. While traditional prognostic markers such as estimated glomerular filtration rate (eGFR) and albuminuria provide valuable insights, they may not fully capture the complexity of CKD progression and associated cardiovascular (CV) risks.

This paper reviews the current state of renal and CV risk prediction in CKD, highlighting the limitations of traditional models and the potential for integrating artificial intelligence (AI) techniques. AI, particularly machine learning (ML) and deep learning (DL), offers a promising avenue for enhancing risk prediction by analyzing vast and diverse patient data, including genetic markers, biomarkers, and imaging. By identifying intricate patterns and relationships within datasets, AI algorithms can generate more comprehensive risk profiles, enabling personalized and nuanced risk assessments.

Despite its potential, the integration of AI into clinical practice faces challenges such as the opacity of some algorithms and concerns regarding data quality, privacy, and bias. Efforts towards explainable AI (XAI) and rigorous data governance are essential to ensure transparency, interpretability, and trustworthiness in AI-driven predictions.

## Introduction

Chronic kidney disease (CKD) is defined by the presence of kidney damage, primarily marked by albuminuria, and/or decreased kidney function (estimated glomerular filtration rate [eGFR] < 60 mL/min/1.73 m^2^), persisting for at least 3 months [1]. It represents a prevalent non-communicable health condition characterized by the gradual loss (or stability in the most favorable situations) of renal function over time. Epidemiological studies have consistently demonstrated a striking prevalence of CKD, with estimates ranging from 9 to 10% within the general population, worldwide. Prevalence and incidence are progressively growing, being almost duplicated over the past decades [2].

The presence of CKD, irrespective of other individual demographic and clinical characteristics such as age, sex, presence of diabetes, hypertension and cardiovascular (CV) disease, exposes patients to an increased risk for future negative health events [3–5]. Considered together, these data revealed that CKD represents an urgent ‘public health’ problem and prompts the improvement investigation of all aspects of disease, namely etiology, risk factors, and optimal management strategies. Understanding the epidemiological landscape of CKD, indeed, is pivotal for informing healthcare policies, allocating resources, and initiating interventions aimed at reducing its burden and improving patient outcomes on a global scale**.**

Chronic kidney disease is characterized by a variety of complications (i.e. hypertension, mineral bone disorders, hyperkaliemia, albuminuria, anemia) that, when combined, have the potential to significantly alter the individual patients’ prognosis. All these variables can present in CKD patients especially when levels of kidney function (eGFR) significantly decrease but their prognostic weight is not the same in all affected patients. The most important variables investigated in terms of prognosis of CKD are eGFR and albuminuria (the abnormal presence of albumin in urine), the so-called ‘kidney measures’ that account for most of the prediction power in the currently available models [6]. However, prediction models with acceptable performance, kidney measures only do not fully predict the future risk of CKD progressing to kidney failure (KF), fatal and non-fatal cardiovascular (CV) events and mortality. This is due to the fact that kidney function related risk factors in CKD patients do not uniformly contribute to the abovementioned future events. Moreover, not all CKD patients respond with the same magnitude to the nephroprotective treatment and thus a significant portion of patients remain at high CV and renal risk under even the best possible care [8–10]. One reason for this low reliability in prediction is the complexity and heterogenicity of mechanisms underlying kidney damage with several factors playing variable roles. Strategies to face such complexity are represented by the construction of prediction models that allow to “adjust” for a multitude of variables, also called “confounders”. Another strategy is to furtherly simplify the information by creating risk scores in which point of risks can be assigned to each variable (e.g. 1, 2, or 3 points to increasing categories of albuminuria < 30, 30–300, > 300 mg/g depending on the predicted risk). The emergence of artificial intelligence (AI) in the past few years may represent an opportunity to improve risk prediction in CKD. Artificial intelligence already showed great potential in several fields of medicine [11, 12].

The aim of the present article is to review the state of art of renal and CV risk prediction in CKD and to highlight how AI can provide simple and complex answers to clinical and research questions.

## Prognosis in chronic kidney disease patients

The association between CKD and a future worse prognosis is an interesting matter of discussion [13]. In fact, the mechanisms underlying the abovementioned associations involve a series of pathophysiological processes. Proteinuria often signifies renal damage and inflammation, while a low eGFR reflects reduced renal capacity to filter and eliminate metabolic waste products. It was also demonstrated that proteinuria is a systemic marker of CV and endothelial damage, according to the ‘Steno hypothesis’, other than a marker of kidney disease itself. Both these factors, proteinuria and eGFR, can contribute to the progression of kidney disease and increased cardiovascular risk through shared processes such as endothelial dysfunction, activation of the renin–angiotensin–aldosterone system, and systemic inflammation. Understanding and managing these pathological mechanisms are crucial for the prevention and optimal treatment of CKD and its cardiovascular and renal complications.

Including the kidney measures, namely eGFR and proteinuria confers several advantages in predicting mortality risk, ESKD, and CV events compared to traditional risk factors and monitoring these factors over time may help to relent CKD progression and to reduce CV risk. This approach enables more personalized risk management [15–16].

The association between proteinuria, eGFR, and the risk of mortality, ESKD, and cardiovascular events has been proved in different population settings. Several studies have highlighted that both a reduction in eGFR and an increase in proteinuria are strongly correlated with an elevated risk of mortality and the development of ESKD. Specifically, an eGFR below 15 ml/min per 1.73 m^2^, below the threshold of 45 ml/min per 1.73 m^2^, has been associated with a significantly increased risk of mortality (hazard ratio (HR): 1.47, *p* = 0.000) and ESKD (HR: 6.24, *p* < 0.001), while an eightfold higher albumin or protein–creatinine ratio has been correlated with a greater risk of mortality (HR: 1.40, *p* = 0.101) and ESKD (HR: 3.04, p < 0.001). These associations have been found both in the general population and in high-risk populations, such as diabetic patients or those with pre-existing cardiovascular diseases [17].

Furthermore, it has been demonstrated that both reduced eGFR and the presence of proteinuria are independent (non-traditional) CV risk factors. They have about a similar prognostic weight of that provided by the sum of ‘traditional’ risk factors, such as hypertension, sex, smoking habit, serum cholesterol levels.

Other important risk factors influence prognosis in CKD patients. Among these, as complications of CKD itself, we remember: anemia, hyperkaliemia, metabolic acidosis, hypertension and hyperphosphatemia.

Anemia, defined as hemoglobin (Hb) < 13.0 g/dl for men and < 12.0 g/dl for women, is a common complication of CKD. It has multifactorial causes like EPO deficiency, uremic-induced inhibitors of erythropoiesis, reduced erythrocyte survival, disordered iron homeostasis and chronic inflammation [18]. The negative effects on health and development outcomes result from the impacts of decreased oxygen delivery to tissues, so multiple organ systems may be affected [19]. The prevalence increases with CKD stage. In the NHANES analysis, it was 17.4%, 50.3%, and 53.4% in stages 3, 4, and 5 CKD, respectively. Based on observational data, anemia is associated with an increased risk of cardiovascular events, all-cause mortality and CKD progression [20]. In fact, it was shown, for every 1-g/dL decrease Hb concentration, there is 11% greater chance of time to dialysis therapy and doubling of serum creatinine concentration. The most emblematic relationship between anemia and heart disease is the development of left ventricular hypertrophy (LVH). LVH is strongly and independently associated with increased mortality risk [21].

Hyperkalemia, defined as a serum potassium > 5.0 mmol/L, is one of the most frequent electrolyte abnormalities in CKD as result of reduced renal potassium excretion, renin–angiotensin–aldosterone system (RAAS) inhibitors and mineralocorticoid receptor antagonists (MRA). When the eGFR decreases from 90 to < 20 mL/min/1.73 m2 or stage 5, the prevalence of hyperkalemia increases from 2 to 42% and to 56.7%, respectively [22]. This can lead to changes in muscle function including the heart muscle, and cause arrhythmia. Persistent HK results in poorer clinical outcomes, which are evident from the early stages 1-3a of CKD. Hyperkalemia has consistently been associated with an increased risk of adverse events (mainly all-cause and cardiovascular mortality) in patients with CKD [23].

Metabolic acidosis in CKD is diagnosed in patients with plasma or venous blood bicarbonate concentration < 22 mmol/L. It occurs in about 20% of patients with CKD and increases with the stage of CKD. The causes of metabolic acidosis in these patients are insufficient production of bicarbonate in distal tubule cells in the process of ammonia genesis, disturbed secretion of protons (as titratable acidity) in the proximal and distal tubules, and impaired reabsorption of bicarbonate in the kidney tubule. With serum bicarbonate concentration < 22 mmol/L, the progression of CKD is about 3 times more pronounced than in patients with serum bicarbonate concentration > 26 mmol/L. Furthermore, even if less studied, metabolic acidosis shows to be linked to cardiovascular disease [24].

Hypertension, defined as a systolic BP > 140 mm Hg or a diastolic BP > 90 mm Hg, is a common comorbidity in patients with chronic kidney disease (CKD). The prevalence ranges from 60 to 90% depending on the stage of CKD and its cause. The mechanisms of hypertension in CKD include volume overload, sympathetic overactivity, salt retention, endothelial dysfunction, and alterations in hormonal systems that regulate blood pressure (BP). Chronic kidney disease (CKD) is both a common cause of hypertension and CKD is also a complication of uncontrolled hypertension. It is associated with an increased risk of cardiovascular morbidity and mortality [25, 26].

Worsening of kidney function as a consequence of an elevated BP is evident by a direct relationship between relative risk of developing end-stage kidney disease (ESKD) and BP severity [27].

Hyperphosphatemia occurs rather late in the process of CKD progression, usually from stage 4. Excessive retention of phosphate can cause a wide range of conditions, such as vascular calcifications, impaired bone mineralization, and dysregulated cell signaling and cell death [28]. Several studies found an association between phosphate, even in the normal range, and all-cause mortality, cardiovascular events, or cardiovascular mortality, which showed a 35 percent increase in mortality per mg increase in phosphorus above normal values (95% CI 1.16–1.57). It affect, also, CKD progression and time to ESKD [29]. All these risk factors impose tailored approaches to prevent and treat all CKD complications [30].

To date, prognostic models on kidney disease were built using the risk of ESKD over time. The principal applied score of 2 and 5 year risk of ESKD is the Kidney Failure Risk Equation (KFRE) [6]. The KFRE included age, gender, race, albuminuria and eGFR and demonstrated a good statistical power (c-statistic of about 0.9). Other risk factors of future events such as arterial hypertension, anemia, serum phosphate, calcium and potassium, smoking habit, body mass index and the presence of diabetes have been included in model prediction, but they did not provide sufficient evidence to be considered for large population screening and assessment [31, 32]. The topic is even more intriguing if we consider that a striking number of CKD patients remain, despite the optimal treatment, having high proteinuria and low eGFR and thus at high CV an ESKD risk. Hence, optimizing risk stratification with modern tools can definitely help to make progress in the future management of CKD patients.

## Prognostic models in CKD

The development of prognostic models for CKD patients has seen a good implementation in the past decade, with the development of increasingly sophisticated and accurate tools to predict the course of the disease and associated clinical outcomes. In particular, the main prognostic models in CKD are oriented toward the identification of patients with high-risk for progression to ESKD, CV fatal and non-fatal events and mortality. Usually, the available models integrate a series of different variables that have a plausible biological association with the aforementioned outcomes and that can play a prognostic effect even after “adjusting” for each other in the model. Variables included in the models, that are so called multivariable models, can be clinical, laboratory and demographic. These may include age, sex, race, blood pressure, eGFR levels, proteinuria, presence of diabetes mellitus, hemoglobin, serum phosphorus levels and other risk factors [15, 33, 34].

To date, prognostic models for CKD are mainly based on the so-called “frequentist approach”. Such an approach is based on the concept that when studying the population, sampling from the population introduces uncertainty. Statistical inference provides us with tools to quantity this uncertainty by answering the question “what would happen if I repeated the study many times?”. In fact, for each estimator of frequentist approach we can compute a confidence interval (usually at 95%) that informs us about the variability of our average value if we repeat the test. And this is applicable to the mean, the odds ratio, the hazard ratio and any other measure derived from the frequentist approach [35].

The study design which is more adaptive to evaluate prognosis in epidemiologic application to CKD is the cohort study, designed to evaluate the role of a certain factor on future events (in terms of temporal sequence) not present at the first evaluation of patients.

Cohort studies are fundamental study designs that follow a group of individuals over time to assess the association between risk factors and the development of diseases or events. They involve the prospective monitoring of groups exposed and not exposed to risk factors, allowing for comparison of the risk of developing the event of interest and identifying causal associations. These studies lead to the identification of risk factors associated with CKD progression, such as hypertension, diabetes, albuminuria, and are also used to assess the effectiveness of a certain treatment in slowing down the progression of the disease.

Kaplan––Meier estimator and Cox proportional hazard models are the principal tools implemented in the context of cohort studies to assess prognosis [23, 36]. The Kaplan–Meier method can be used to estimate the cumulative survival of patients with CKD over time, considering events such as disease progression, dialysis, or kidney transplantation. This method allows for the assessment of the survival probability of CKD patients and for comparing survival curves among subgroups of patients with different risk levels. One significant limitation of the Kaplan–Meier analysis is its allowance for only “crude” risk, namely univariate analysis, which does not account for variables (also known as “confounders”) that may influence the association of exposure with the event. Overcoming this limitation is made possible by the Cox proportional hazards regression. Cox regression is a semi-parametric statistical method used to analyze survival data and assess the effect of independent variables on the risk of an event over time. One of the main measures resulting from the Cox model is the hazard ratio (HR), which represents the instantaneous ratio between the risks of two groups of individuals with different characteristics regarding a variable of interest [37]. The Hazard Ratio provides information on the magnitude and direction of the effect of a variable on the probability of an event occurring over time. Essentially, the Hazard Ratio calculated from the Cox model allows for evaluating the association between a predictive variable and the outcome of interest, providing an estimate of the relative risk (RR) of developing the event in the exposed group compared to the reference group. In the context of CKD, the Cox model enables the identification of the most significant risk factors influencing the progression of kidney disease, assessing the effectiveness of management strategies, and developing personalized interventions to improve patients' clinical outcomes [38]. One variant of the Cox model is the Fine and Gray model [38, 39]. The Fine and Gray model shares some features with the Cox model but is able to compute a relative risk of an event during time, accounting for the competing risk of a second event (e.g. mortality and ESKD in CKD patients) that can occur in a competitive fashion. This type of risk is provided as sub-hazard ration and is gaining momentum in assessing prognosis of CKD patients, especially those with high CV risk at baseline. The respective of Kaplan–Meier curves in the competitive risk scenario is represented by the cumulative incidence, also known as Aalen-Johansen estimator [40, 41]. The cumulative incidence, unlike Kaplan–Meier estimator that ignores the competing event, adjusts the slope of the curve and thus the probability of events after calculating the risk (for each category of patients) of the competing event of interest. One example of risk prediction, in patients with CKD, using the competing risk approach is represented by the novel KD predict [42]. This score computes the 1 (up to 5)-year risks of death and kidney failure by accounting that both are competing events. For example, according to KFRE a 50 year old female with CKD, eGFR of 32 mL/min/1.73m^2 ^and urine albumin-to-creatinine ratio (ACR) of 150 mg/g had a 5-year ESKD risk probability of 12.48% whereas the same probability for the same patient was 11% only with ‘KD predict’ tool. To date, cohort studies and the classical inference estimators are the principal tools used to study the principal outcomes of CKD patients. The main limitations of classical tools are that they provide the risk with a limited number of variables (the investigator should know the variable of interest) and that risk is manifested at a certain moment in time. Moreover, these models provide an average increased risk for each variable, namely a 30% increased risk to CV events in presence of severe proteinuria as compared with moderate or normal proteinuria. But, computing the true risk considering multiple parameters, and possibly the most significant, is often complex with traditional methods. Novel tools, including AI, may intervene in discovering unknown risk factors and in refining the combination of old and novel risk factors to improve with greater accuracy the event prediction.

## Artificial intelligence in chronic kidney diseases prediction and management

It is nontrivial to furnish a definition of Artificial Intelligence (AI) due to the inherent complexity in defining the concept of intelligence itself. Alan Turing's landmark paper in 1950 established a test, known as *imitation game*, for assessing machine intelligence, proposing that for a machine to be considered intelligent, it must respond in ways indistinguishable from human behavior. Consequently, Artificial Intelligence is acknowledged as the capacity of a machine or a software to perform tasks necessitating human intelligence. Despite the large number of techniques included, one approach known as Machine Learning (ML) has gained undisputed fame. Machine learning can be seen as a method to enable a machine or a program to acquire that ability through a *training* process. According to Tom M. Mitchell [43], a computer program is said to learn from experience *E* with respect to some class of tasks *T* and performance measure *P*, if its performance at tasks in *T*, as measured by *P*, improves with experience *E*.

Machine learning techniques can be broadly categorized into supervised learning and unsupervised learning.

The former, involves training a model on labeled data, where the outcome (target variable) is known. This category includes both classification and regression techniques, based on the need to predict categorical outcome (e.g., yes/no, disease/no disease) or continuous outcome, respectively.

The second one involves training a model on data without labeled outcomes. The goal is to uncover hidden patterns or structures in the data (e.g., clustering or dimensional reduction techniques).

Examples of Machine Learning techniques are *linear regression, logistic regression, Lasso, Ridge, support vector machines (SVM), k-nearest neighbors, decision trees, ensembles (e.g., random forest, XGBoost), k-means clustering, principal component analysis (PCA)* and others.

Lasso (Least Absolute Shrinkage and Selection Operator) regression is a type of linear regression that uses L1 regularization. This technique not only minimizes the residual sum of squares between the observed and predicted values but also adds a penalty proportional to the absolute value of the coefficients. This penalty can shrink some coefficients to zero, effectively performing variable selection.

Ridge regression is a type of linear regression that uses L2 regularization. This technique adds a penalty proportional to the square of the magnitude of the coefficients to the residual sum of squares. The penalty term discourages large coefficients, thereby addressing issues of multicollinearity and improving the model’s generalization.

Decision trees are versatile supervised learning techniques used for both classification and regression tasks. They model decisions and their possible consequences as a tree-like structure. A decision tree consists of nodes (i.e., decision points) and branches (i.e., outcomes). The root node represents the entire dataset, which is recursively split based on feature values. At each node, the data is split based on a feature that results in the best separation according to a certain criterion (e.g., Gini impurity, information gain for classification; mean squared error for regression).The terminal nodes (leaves) represent the final decision or prediction [44].

XGBoost is an ensemble of weak models (e.g., decision trees) based on the gradient boosting framework. It constructs additive models by sequentially fitting a weak learner (usually a decision tree) to the residuals of the model from the previous iteration. The boosting process starts with an initial prediction, then at each iteration fit a new tree to the residual errors (i.e., the difference between the actual and predicted values). The new tree is added to the ensemble to reduce the residuals. The final model is the combination of all the individual trees. XGBoost includes regularization terms to control the complexity of the model, preventing overfitting like L1 (Lasso) and L2 (Ridge) regularization [45].

The aforementioned machine learning techniques offer significant advantages over traditional statistical models like the Cox model by not requiring strong assumptions such as proportional hazards or linearity of covariate effects. Their flexibility in handling high-dimensional data and complex relationships allows them to outperform classic models, particularly in scenarios where the data structure and relationships are intricate and do not conform to the assumptions of traditional statistical methods.

Deep Learning (DL) is a subset of machine learning that employs *artificial neural networks* with multiple layers (hence the term "deep") of interconnected nodes called *neurons*, to learn intricate patterns and representations from vast amounts of data. It is inspired by the structure and function of the human brain's biological neural networks. The latter, composed of billions of interconnected neurons, exhibit complex information processing, learning, and adaptation abilities. Deep learning attempts to mimic this behavior using artificial neural networks where each neuron receives inputs, applies a transformation using weights and biases, and produces an output that serves as input to the neurons in the next layer. In fact, neural networks are generally organized into layers. The input layer receives the input data, then there is a stack of multiple hidden layers that perform transformations on the data, finally an output layer which produce the final output. During the training process, the “Backpropagation” is performed for each training pace. It calculates the gradients of the loss function with respect to each weight and bias, propagating errors backward through the network and updating the weights and biases to reduce the loss. The loss function measures the difference between the network’s predictions and the actual values. Common loss functions include Mean Squared Error (MSE) for regression and Cross-Entropy for classification.

**Fig. 1:**
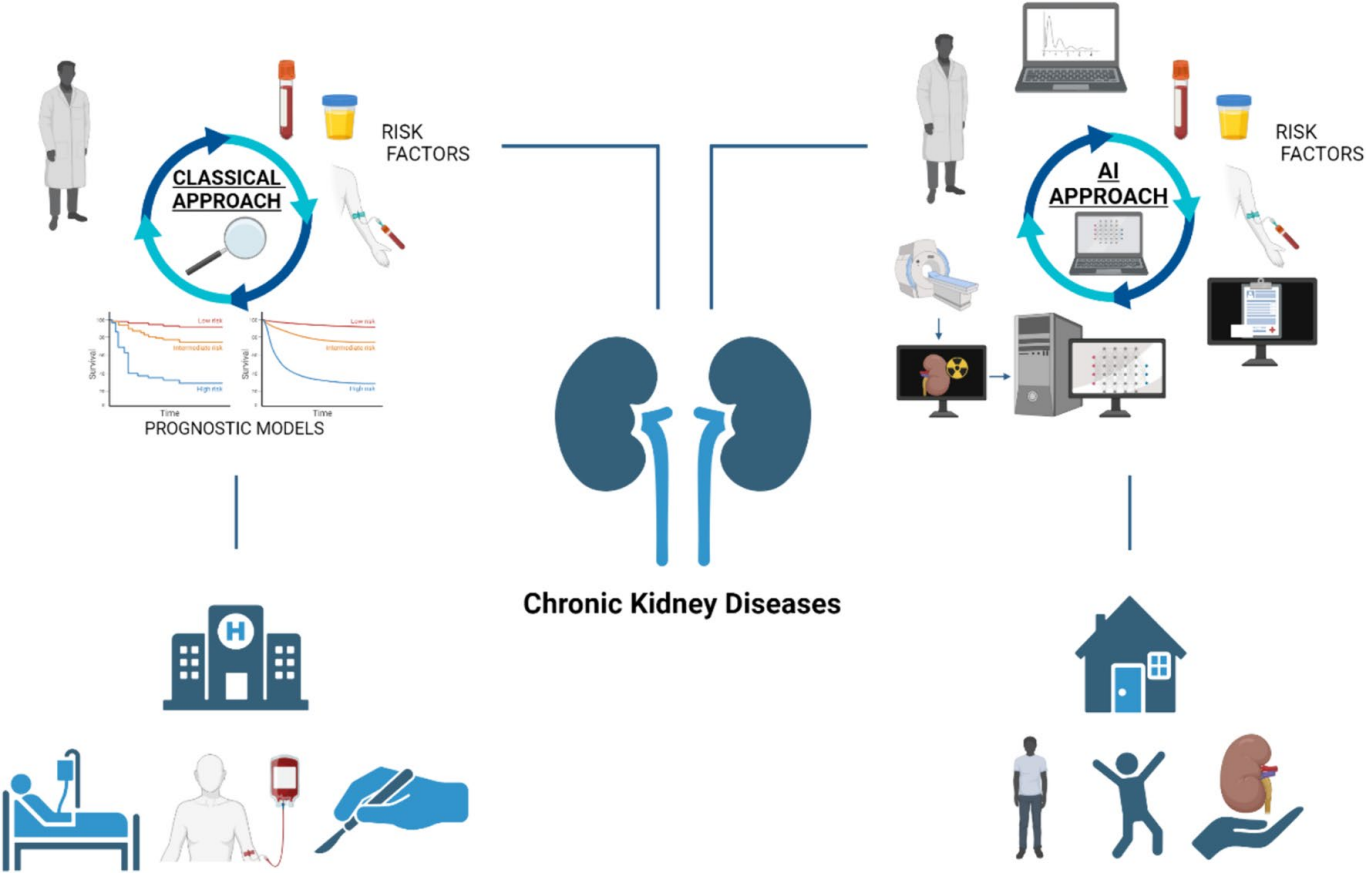
Applications of artificial intelligence to clinical approach of CKD patients

The integration of artificial intelligence into medical research and practice is showing a rapidly growing trend powered by the increasing availability of healthcare digital data. Common applications include performing early diagnosis, improving risk prediction and management, improving communication between patient and specialist, drug discovering, mental health issues identification [46] and others (Fig. 1).

Furthermore, in the last decade, several AI-based devices have been approved by the Food and Drug Administration (FDA)^1^ and a lot of them have been successfully implemented.

Due to the lack of education in digital medicine, several countries, including Italy, have introduced a pioneering university degree program. One notable institution implementing this initiative is UniCal with its MEDTD^2^ (Medicine and Digital Technologies) course. This program integrates medical and computer engineering curricula, aiming to equip doctors with the skills to understand and apply the latest techniques to traditional medicine.

In the context of CKD, where an accurate and an early prediction of renal and cardiovascular (CV) risk is paramount for effective management, AI-based approaches hold significant potential.

One of the primary advantages of employing AI in CKD research lies in its ability to analyze vast amounts of patient data and identify complex patterns that may not be readily apparent to human observers. Traditional prediction models for CKD prognosis rely heavily on kidney measures such as estimated glomerular filtration rate (eGFR) and proteinuria, which provide valuable insights but may not capture the full spectrum of risk factors contributing to disease progression and adverse outcomes. AI algorithms can complement these measures by integrating additional information, such as genetic markers, biomarkers, comorbidities, socio-economic factors, as well as bioimages (e.g., pictures, CT and ultrasound) to generate more comprehensive risk profiles for individual patients. For instance, according to [47] deep-learning models can be used to identify chronic kidney disease and type 2 diabetes solely from retinal fundus photographs in combination with clinical metadata (age, sex, height, weight, body-mass index and blood pressure), while in [48] deep-learning models trained instead on external photographs of the eyes can be used to detect diabetic retinopathy (DR), diabetic macular oedema and poor blood glucose control.

Furthermore, AI-based approaches offer the flexibility to adapt and refine predictive models over time as new data becomes available, enabling continuous improvement in accuracy and performance. Deep learning algorithms, in particular, excel at extracting hierarchical representations from complex datasets, allowing for more nuanced and personalized risk assessments in CKD patients [49]. By leveraging techniques such as convolutional neural networks and recurrent neural networks, deep learning models can discern subtle relationships between disparate variables and uncover novel predictive insights. A summary of the main paper concepts is reported in Table 1.

**Table 1:** Summary of the main paper results

	Key-messages
Prognosis in chronic kidney disease patients	CKD is characterized by a progressive decline in renal function, marked by decreased eGFR and the presence of proteinuria. These indicators are crucial as they are associated with increased risks of mortality, end-stage kidney disease (ESKD), and cardiovascular (CV) events. This holds true across both general and high-risk populations, such as those with diabetes or previous CV events
	The associations between CKD indicators and adverse outcomes involve various pathophysiological processes. Proteinuria signifies renal damage and systemic inflammation, while low eGFR indicates reduced renal filtration capacity. Both factors contribute to CKD progression and elevated CV risk through mechanisms like endothelial dysfunction, activation of the renin–angiotensin–aldosterone system, and systemic inflammation
	Including eGFR and proteinuria in prognostic models enhances the prediction of mortality, ESKD, and CV events compared to traditional risk factors alone. Monitoring these variables over time facilitates personalized risk management, potentially slowing CKD progression and reducing CV risks. The Kidney Failure Risk Equation (KFRE) is a notable model that integrates these factors and shows strong predictive power
	Both reduced eGFR and proteinuria are independent CV risk factors, with prognostic significance comparable to traditional risk factors like hypertension, gender, smoking, and cholesterol levels. Despite optimal treatment, many CKD patients remain at high risk due to persistent proteinuria and low eGFR, underscoring the need for improved risk stratification and management strategies to enhance patient outcomes
Standard prognostic model	There has been notable progress in developing sophisticated and accurate prognostic models for CKD. These models aim to predict disease progression, the onset of ESKD, CV risks, and mortality by integrating a diverse range of clinical, laboratory, and demographic variables
	CKD prognostic models typically employ multivariable approaches, incorporating factors such as age, sex, race, blood pressure, eGFR, proteinuria, presence of diabetes, hemoglobin levels, and serum phosphorus levels. These models adjust for interactions between these variables to improve the precision of risk predictions
	Cohort studies, which track groups of individuals over time, are essential for identifying risk factors linked to CKD progression. The Kaplan–Meier estimator is used to estimate cumulative survival, while the Cox proportional hazard model assesses the impact of various risk factors on the likelihood of an event, providing a measure known as the Hazard Ratio (HR)
AI in medical research and practice	AI’s integration into medical research and practice is rapidly growing due to the increasing availability of healthcare digital data. AI applications in healthcare include early diagnosis, risk prediction and management, patient-specialist communication, drug discovery, and mental health issues identification
	Several AI-based medical devices have been approved by the Food and Drug Administration (FDA)^a^ and implemented successfully
	Due to the need for education in digital medicine, countries like Italy have introduced university degree programs integrating medical and computer engineering curricula (e.g., University of Calabria, Italy, with the UniCal's MEDTD^a^ (Medicine and Digital Technologies) course)
AI in chronic kidney disease (CDK) research	AI holds significant potential in CKD research for early and accurate prediction of renal and cardiovascular risk allowing for personalized risk assessments in CKD patients
	AI can analyze vast amounts of patient data and identify complex patterns beyond traditional prediction models, through the integration of additional information (genetic markers, biomarkers, comorbidities, socio-economic factors, bioimages) to create comprehensive risk profiles
	Deep-learning models can identify CKD and type 2 diabetes from retinal fundus photographs in combination with clinical metadata (age, sex, height, weight, body-mass index and blood pressure) [47] and external photographs of the eyes can be used to detect diabetic retinopathy (DR), diabetic macular oedema and poor blood glucose control [48]
Explainable AI (XAI) in clinical practice	AI-based approaches in clinical practice face significant challenges, particularly the “black box” nature of deep-learning models which limits their interpretability and the ability for clinicians to understand their predictions. Indeed, due to their complexity, deep learning models often do not allow for tracing the path from input to output, making it difficult to understand how decisions are made
	Researchers are increasingly focusing on Explainable AI to address these issues. XAI aims to make AI models more transparent by providing interpretable explanations for their decisions. Techniques, such as feature importance analysis, attention mechanisms, and model-agnostic approaches are used in XAI to uncover the factors driving AI predictions
	Enhancing transparency helps clinicians trust AI outputs, assess their reliability, and integrate them into clinical decision-making processes more confidently. Addressing the black box problem is essential for ensuring accountability, compliance with regulatory standards, and addressing concerns regarding patient safety, privacy, and ethical implications

^a^https://www.fda.gov/medical-devices/software-medical-device-samd/artificial-intelligence-and-machine-learning-aiml-enabled-medical-devices

^a^https://offertaformativa.unical.it/lt-lmcu/cds/0822-medicina-e-chirurgia-td/brochure/

## Artificial intelligence: challenges to address and pitfalls

However, despite the potential advantages of AI-based approaches, several pitfalls and challenges must be addressed to ensure their effective integration into clinical practice. One notable concern is the "black box" nature of some AI algorithms which may obscure the rationale behind their predictions and limit interpretability for clinicians. In particular, due the complexity of the structure of deep learning models, it is usually impossible to know why they return an output given a specific input because it is not possible to follow the full path from the input to the output. Nonetheless, in recent years, researchers are focusing on the Explainable AI (XAI) approach to mitigate this issue. Explainable AI aims to enhance the transparency of AI models by providing interpretable explanations for their decisions, thus enabling clinicians to understand and trust the outputs generated by these algorithms. Through techniques such as feature importance analysis, attention mechanism, and model-agnostic approaches, Explainable AI seeks to uncover the underlying factors driving AI predictions [50]. Feature importance analysis calculates a score for all the features included in a model, so that the larger is the score the larger is the predictive effect of a certain feature. The Attention mechanism is a technique used to improve the model performance by assigning to features a weight to each feature. These weights determine the level of importance of the feature in the model. Interestingly, model agnostic approach is a methods that aims at interpreting the predictive response of a black box model rather than the response of the original data [51]. By gaining insights into the decision-making process of AI systems, clinicians can better assess the reliability of AI-generated recommendations and incorporate them into their clinical decision-making process more confidently. Additionally, addressing the black box problem is crucial for ensuring accountability and compliance with regulatory standards in healthcare, as opaque algorithms may raise concerns regarding patient safety, privacy, and ethical implications. Therefore, while the inherent opacity of some AI algorithms poses a significant challenge, the emergence of Explainable AI methodologies offers promising solutions to promote the responsible and effective integration of AI in clinical settings.

Ensuring transparency and explainability in AI models is crucial for building trust and facilitating their adoption in real-world healthcare settings. Artificial intelligence may be involved in several steps of clinical decision making in nephrology, from diagnosis to treatment decision (based on the early prediction of response to treatment) and to an improvement of routine clinical procedures (e.g. outpatients evaluation and kidney transplant list assessment).

## Conclusions

In summary, the application of AI in CKD research holds promise for enhancing risk prediction and personalized management strategies. By harnessing the power of machine learning and deep learning techniques, researchers and clinicians can unlock new insights into the complex dynamics of CKD progression and improve patient outcomes. However, it is essential to address the challenges posed by AI adoption and ensure responsible and ethical use of these technologies in clinical decision-making.

## Data Availability

No datasets were generated or analysed during the current study.

## Footnotes

1 https://www.fda.gov/medical-devices/software-medical-device-samd/artificial-intelligence-and-machine-learning-aiml-enabled-medical-devices.

2 https://offertaformativa.unical.it/lt-lmcu/cds/0822-medicina-e-chirurgia-td/brochure/.
